# Not All Slugs Are the Same: Variation in Growth and Development of the Slug *Deroceras reticulatum*

**DOI:** 10.3390/insects11110742

**Published:** 2020-10-29

**Authors:** Mark Shirley, Sally Howlett, Gordon Port

**Affiliations:** 1 School of Natural and Environmental Sciences, Newcastle University, Newcastle upon Tyne NE1 7RU, UK; mark.shirley@newcastle.ac.uk (M.S.); sally.howlett@york.ac.uk (S.H.); 2 Department of Biology, University of York, Heslington, York YO10 5DD, UK

**Keywords:** population ecology, slug population, growth, development, *Deroceras reticulatum*

## Abstract

**Simple Summary:**

Slugs are important pests. Models to forecast slug populations make assumptions about growth and mortality in response to environmental factors. To refine these models, we studied the effect of hatching season on the growth and survival of *Deroceras reticulatum*, a worldwide pest. Slugs hatching in spring and autumn were compared at three rearing temperatures (ambient, 12 °C and 15 °C). *Deroceras reticulatum* reared under identical conditions showed great variation in growth and strong bimodality in growth rates. At all rearing temperatures, growth was influenced by hatching season; in all cases, fast growers dominated in autumn and slow growers dominated in spring. *Deroceras reticulatum* populations may be partitioned into ”slow growers” and ”fast growers”. Fast growers responded to warmer conditions, growing to large sizes. Slow growers, in contrast, gained weight at comparable rates to ambient reared slugs, regardless of the elevated constant temperatures. The peaks of slug activity seen in the field in autumn and spring are possibly not distinct generations as some slugs may mature early/late and slip into the alternative cohort. Rather, the observed autumn and spring peaks in slug numbers may be a response of a mixed-age population to the favourable environmental conditions at that time.

**Abstract:**

Models to forecast slug populations make assumptions about growth and mortality in response to environmental factors. To refine these models, the growth trajectories and survival of *Deroceras reticulatum*, a worldwide pest, hatching in spring and autumn were compared at three rearing temperatures (ambient, 12 °C and 15 °C). *Deroceras reticulatum* reared under identical conditions showed great variation in growth and strong bimodality in growth rates. At all rearing temperatures, growth was influenced by hatching season; in all cases, fast growers dominated in autumn and slow growers dominated in spring. Survival was influenced by hatching season: autumn-born slugs survived better at ambient temperatures, but spring-born slugs had better survival at 15 °C. *Deroceras reticulatum* may be partitioned into ”slow growers” and ”fast growers”. Fast growers responded to warmer conditions, growing to large sizes. Slow growers, in contrast, gained weight at comparable rates to ambient reared slugs, regardless of the elevated constant temperatures. The peaks of slug activity in autumn and spring are possibly not distinct generations as some slugs may mature early/late and slip into the alternative cohort. Rather, the observed autumn and spring peaks in slug numbers may be a response of a mixed-age population to the favourable environmental conditions at that time.

## 1. Introduction

Underpinning effective slug control is the need to predict when and where particular increases in populations are likely to occur so that steps can be taken in advance to minimise the economic loss that would otherwise result [1]. Various models have been proposed to forecast changes in slug population dynamics as a consequence of environmental factors and their impact on growth and mortality [2,3,4]. Due to the complexity of slug behaviour and limited data on some aspects of their fundamental biology, however, these are often based on a number of assumptions about lifecycle parameters and have, to a greater or lesser extent, only been able to predict confidently part of the overall picture. There is a need, therefore, for studies to bridge these gaps in the knowledge of slug biology so that models can give more accurate predictions.

The potential of a slug population to attain pest proportions is largely determined by its initial size and the speed with which it can complete its lifecycle whilst favourable conditions prevail. It is well-established that temperature influences many aspects of the biology of terrestrial slugs, including growth rate [5,6,7]. For *Deroceras reticulatum*, most studies have shown that the relationship between growth rate and temperature is approximately hyperbolic; there is a positive association up to an optimum of 17–19 °C after which higher temperatures have a detrimental effect on development [8,9,10,11]. An exception to this are the works of Judge [12] and Clemente et al. [13] whose data indicate that growth is faster at cooler temperatures. All of these studies are based on slugs hatching at one particular point in time—i.e., a single season—although few actually state which season this is. *D. reticulatum* is capable of breeding throughout the year if conditions are favourable, but there are peaks in spring and autumn [14,15,16]. The autumn population has to over-winter, either as eggs or recently hatched juveniles, and it is postulated that there may be something inherently different in the physiology of these slugs compared to the spring population that adapts them for development and survival at lower seasonal temperatures. If so, it might be expected that the growth trajectories of spring and autumn hatching slugs will differ when reared under identical conditions. This may explain the discrepancy between the work of Judge [12], Clemente et al. [13] and other authors.

There are no published data that directly assess the effect of hatching season on slug growth. The experiments presented in this paper were, therefore, designed to investigate this. Our first hypothesis is that hatching season will affect slug growth trajectory under otherwise identical conditions. Our second hypothesis is that survival will be affected by hatching season. The growth trajectories of slugs hatching in spring and autumn were compared at three rearing temperatures (ambient, 12 °C and 15 °C), along with their survival. It was also possible to contrast growth and survival between temperatures within a given season to see how trends compare with published studies. In addition to finding out more about the biology of *D. reticulatum*, the results of this paper may be of practical application in refining population dynamics models used in risk assessments for the control of slugs.

## 2. Materials and Methods

### 2.1. Experimental Procedure

Two identical studies were completed—one in autumn 2002 and one in spring 2003. In each study, eggs used were laid by 50 field-collected adult *D. reticulatum*. The adults were collected from under refuge traps at Heddon-on-the-Wall, Northumberland (Grid reference NZ 127659). They were weighed using a Mettler MT5 balance, and placed in individual Petri dishes lined with moist laboratory tissue. Their diet consisted of Chinese cabbage and carrot ad libitum, with cuttlefish bone provided as a source of calcium. The slugs were maintained at a constant temperature of 20 ± 2 °C (mean ± S.E.) in a Sanyo MIR-253 incubator with a constant photoperiod of 16:8 L:D and were cleaned weekly by transferring them to a clean dish with fresh food. Over a two-week period, a total of 60 egg batches were collected. Each batch was placed on fine grade netting and rinsed with distilled water to remove any soiling before transfer into another Petri dish lined with moist laboratory tissue. The number of eggs per batch was recorded.

The 60 egg batches were allocated equally and at random to three temperature treatments: two constant (12 ± 2 °C and 15 ± 2 °C) (mean ± S.E.) and one fluctuating (ambient). Constant temperatures were maintained in Sanyo MIR-235 incubators with a photoperiod of 16:8 L:D, provided by two 15 W fluorescent tubes. The temperatures were monitored using Tinytalk^®^ data loggers (Gemini Data Loggers, UK). For the ambient treatment, Petri dishes containing egg batches were placed in a plastic tank housed outside. The temperature inside and outside the tank was also recorded using a Tinytalk^®^ data logger, and there was no additional light (i.e., natural photoperiod).

Egg batches were prevented from drying out by remoistening with distilled water as required. Hatching was checked weekly. Regular monitoring continued until two full weeks had elapsed since the last slug hatched. On each occasion, any offspring were removed from the Petri dishes and entered into the next stage of the study.

At each of the three rearing temperatures for a given season, a total of 200 individuals were initially monitored. Due to very low mortality rates in all treatments, this number was subsequently reduced to 100 individuals per treatment selected at random in order to make the experiment more manageable. Throughout the experiment, slugs were handled using a square-ended paintbrush. Hatchlings were gently removed from the Petri dishes in which egg batches had been incubated. Each individual was placed into a separate 9 cm diameter Petri dish lined with laboratory tissue moistened with distilled water. Chinese cabbage and carrot were provided ad libitum, with cuttlefish bone as a source of calcium. The slug was then returned to the temperature treatment at which it hatched. Dishes were cleaned weekly when food was replaced. During cleaning, the slug was transferred to the lid of the Petri dish. The moist laboratory tissue was then replaced, and any soiling was wiped from the surfaces of the dish. Fresh food was added, and the slug was transferred back into the dish. Mortality was recorded weekly.

Slugs were weighed at hatching (week 0) and fortnightly thereafter for a total of 20 weeks to an accuracy of 0.01 mg, allowing a brief settling period for the reading to stabilise.

### 2.2. Statistical Methods

Data for comparing slug weights between treatments were tested for normality and transformed if necessary. Survival data were analysed with the Cox proportional hazards, using the log-rank test to compare between treatments. The proportional hazards assumption of the survival analysis was tested with Schoenfeld individual tests.

Slug growth was analysed with a linear model fitted for each slug using Generalised Least Squares (GLS), with slug weight as the response and week as the explanatory variable, with temporal autocorrelation to account for repeated measures of each slug. The model outputs were collated; 90.5% of these models had an R^2^ greater than 0.75. The gradient calculated for each linear model represents the average growth rate of the slug.

A finite mixture model (FMM) was used to investigate the likelihood that the population of individual growth rates comprised two distinct normally-distributed subpopulations with separate means and standard deviations. The Expectation Maximisation algorithm used in FMM sorts the individual data independently of covariates such as hatching season or growth temperature. Each individual was assigned a posterior probability of belonging to each of the two posited subpopulations.

These statistical analysis were performed in R 3.6.0 [17], using the mixtools [18] and nlme [19] packages.

## 3. Results

Parents laying eggs in spring were significantly heavier than those laying eggs in autumn (ANOVA: F1, 67 = 6.266, *p* < 0.001). The mean parental weight (± S.E.) in spring was 585.43 ± 22.73 mg, and in autumn it was 469.10 ± 18.31 mg. The mean egg batch size did not differ between treatments either between or within seasons. The mean ambient temperature (± S.E.) during the hatching period was 13.3 ± 0.1 °C (range 4.3–24.1 °C) in spring and 5.7 ± 0.1 °C (range −3.0–21.8 °C) in autumn.

### 3.1. Growth Analysis

Table 1 shows the numbers of slugs alive for the full 20-week monitoring period in each treatment after numbers were reduced (see methods). All growth analyses are based on these slugs.

The mean (± S.E.) ambient temperature during the 0–20-week growth period was 12.7 ± 0.1 °C (absolute range: 1.2–24.1 °C; mean daily range: ± 4.9 °C) in spring and 5.6 ± 0.1 °C (absolute range: −3.0–14.0 °C; mean daily range: ±2.8 °C) in autumn.

The mean weights of the slugs at week 20 are shown in Table 2. There were marked differences in growth between seasons at a given rearing temperature, but also between some rearing temperatures in a single season, most notably autumn. There were opposite trends between the seasons in the rearing temperatures at which maximum and minimum mean growth was reached; in spring the highest mean weight was reached in ambient conditions and the lowest at 15 °C, whereas in autumn the reverse was observed.

**Figure 1:**
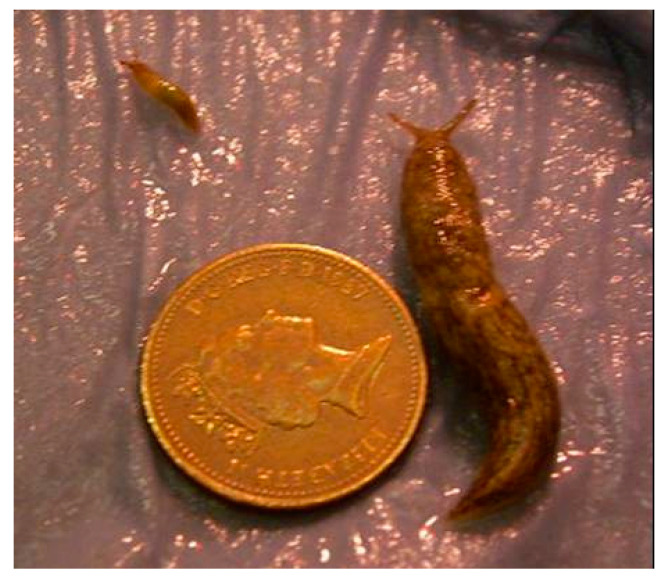
Growth variation in *Deroceras reticulatum* of the same chronological age (20 weeks). Slugs were reared under identical conditions. Both hatched in spring and were maintained at 15 °C. Scale is indicated by a 1 pence piece (diameter 20.3 mm).

**Figure 2:**
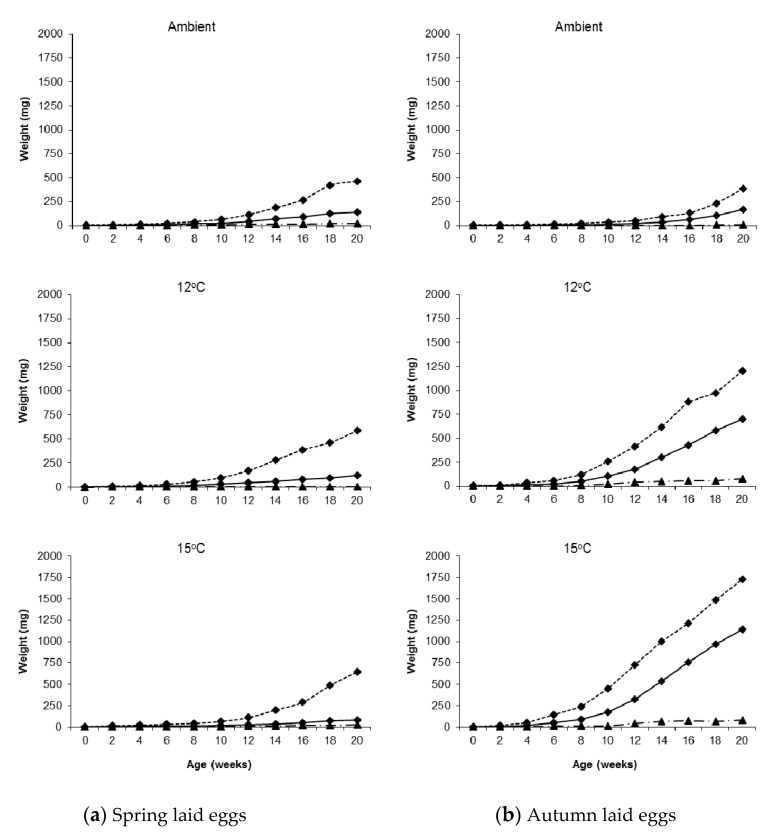
Median (solid line), maximum (dotted line) and minimum (dashed line) weights of *Deroceras reticulatum* plotted against age. Slugs were maintained at ambient temperature, constant 12 °C or constant 15 °C and hatched from eggs laid in (**a**) spring or (**b**) autumn.

Considerable variation in weight was observed in all treatments; Figure 1 shows two spring hatching slugs of the same age reared under identical conditions (15 °C). Twenty weeks after hatching the overall weight range for the largest individuals per temperature in the autumn laid group was 380–1730 mg and for the smallest individuals it was 5–80 mg. For the spring laid group, the ranges were 460–644 mg and 6–25 mg, respectively (Figure 2).

Considering this within treatment variation, there were still significant differences in growth between treatments. At all rearing temperatures, the growth rate from hatching until week 4–5 was similar in both seasons, but then began to diverge, and this became more marked with time. At ambient temperature, slugs hatching in spring grew faster than those hatching in autumn for most of the 20–week monitoring period, although those hatching in autumn began to catch up and overtake in weeks 19–20. At the two constant temperatures, however, the reverse was observed; growth was considerably faster for autumn hatching slugs throughout the monitoring period. By week 20, autumn hatching slugs were on average four times larger than spring hatching slugs at 12 °C, rising to eight times larger at 15 °C.

**Figure 3:**
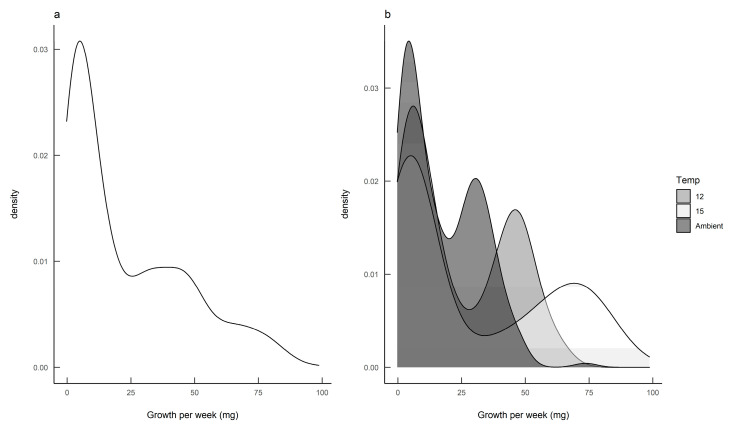
Growth rate analyses for *Deroceras reticulatum* showing (**a**) the density of slug growth data, and (**b**) the density of slug growth data by rearing temperature. Slugs were maintained at ambient temperature, constant 12 °C or constant 15 °C.

The density plot for the calculated growth rates suggested that there were two distinct subpopulations (Figure 3a), and the bivariate nature of the data became more pronounced when the data for each rearing temperature were plotted separately (Figure 3b).

**Figure 4:**
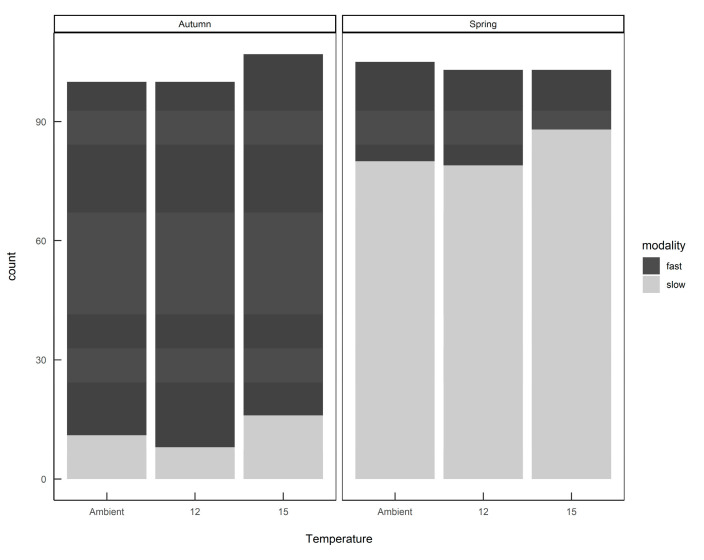
The numbers of *Deroceras reticulatum* exhibiting either fast or slow growth modalities when hatched from eggs in autumn or spring. Slugs were maintained at ambient temperature, constant 12 °C or constant 15 °C.

The finite mixture model (FMM) identified two populations: a slow-growing modality (mean 4.3 mg day^−1^; standard deviation 2.9) and a fast-growing modality (mean 37.8 mg day−1; standard deviation 21.3). Furthermore, these two growth modalities were prevalent in different seasons, with fast-growing slugs predominant in the autumn-hatched individuals and slow-growing slugs predominant in the spring-hatched individuals (Figure 4).

**Figure 5:**
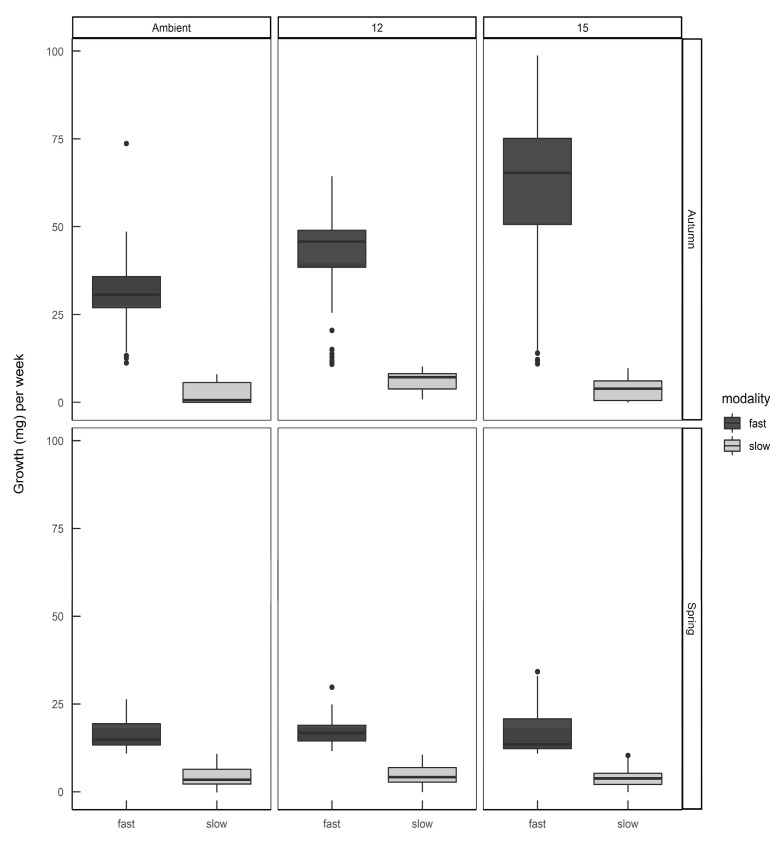
The growth rates of *Deroceras reticulatum* hatched from eggs in autumn or spring and reared at ambient temperature, constant 12 °C or constant 15 °C. Slugs were allocated to fast or slow growth modalities (see text).

When the individual growth rates were plotted by hatching season and rearing temperature (Figure 5) it can be seen that slugs identified as belonging to the ”fast” modality that were hatched in spring still had a lower average growth rate than the same modality hatched in autumn, and rearing temperature had some effect on the distribution of growth rates within each modality. Eggs from the same egg batch developed into slugs of either or both modalities, in proportions representative of the season in which they were laid—there was no evidence that parents laid eggs all of one modality.

### 3.2. Survival Analysis

Table 3 shows the numbers of slugs in the experiment up to week 20, including those that died before the numbers were reduced to 100 per treatment (see methods), hence the numbers exceed 100. All survival analyses are based on these slugs.

The Cox proportional hazards model was used to analyse survival. Schoenfeld individual tests for each survival analysis were all non-significant (Ambient: df = 1, χ^2^ = 1.67, *p* = n.s.; 12 °C: df = 1, χ^2^ = 0.76, *p* = n.s.; 15 °C: df1, χ^2^ = 0.87, *p* = n.s.), suggesting that the assumption of proportional hazards that do not vary with time has not been violated.

**Figure 6:**
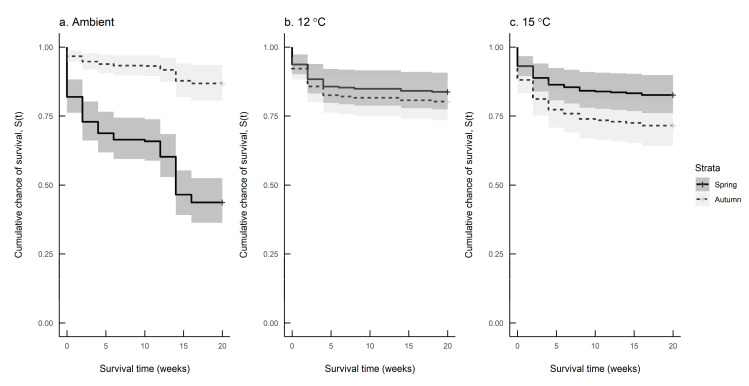
Survival predictions for *Deroceras reticulatum* reared at (**a**) ambient temperature, (**b**) 12 °C and (**c**) 15 °C in spring (solid line) and autumn (dashed line).

Figure 6a–c compare seasonal differences in survival at each of the three rearing temperatures. The survival rate differed significantly between spring and autumn hatching slugs reared at ambient temperature and 15 °C, but not for those reared at 12 °C (Log-rank test: Ambient: *n* = 249, df = 1, χ^2^ = 46.9, *p* < 0.001; 12 °C: *n* = 238, df = 1, χ^2^ = 0.5, n.s.; 15 °C: *n* = 249, df = 1, χ^2^ = 4.2, *p* = 0.04). Table 4 shows the mean survival times of slugs at each combination of hatching season and rearing temperature.

## 4. Discussion

Our results show that slugs may be partitioned into “slow growers” and “fast growers”. Fast growers responded to the warmer than ambient, constant conditions, growing to large sizes. Slow growers, in contrast, gained weight at comparable rates to ambient reared slugs, regardless of the elevated constant temperatures. A consequence of these two growth modalities is that there was considerable variation in the size of slugs at hatching and during subsequent growth, even when reared under identical conditions. Similar size variation has been found for a number of species [7,20,21,22], and such variation is common in pulmonates generally [23]. Since individuals of the same age varied between approximately 5- and 100-fold in their weight and, by inference, slugs of a given weight vary considerably in their age, our results support the conclusion of Prior [21] for *Limax maximus* (L.) that ”one cannot use body weight to estimate the absolute or relative age of animals accurately”. It is clear that the terms ”juvenile” and ”adult” should be used to describe the developmental state rather than the chronological age of slugs.

The mechanisms underlying this exceptional divergence in size are not clear. The variation is unlikely to be adaptively neutral since it is so consistently maintained over time [24]. For example, it allows for the fact that there will be some mature individuals in the population capable of mating whenever conditions are favourable, maximising reproductive opportunities. Shibata and Rollo [22] put forward a number of hypotheses to explain the basis of this phenomenon in *Deroceras laeve* (Müller), a largely self-fertilising species. These included maternal diet and egg quality, “nutritional imprinting” (i.e., influence of early nutritional experience), density effects and egg size. Of these, they found that only egg size had a significant effect on growth with slugs hatching from smaller eggs growing faster than those hatching from larger ones. This was, however, only the case for slugs fed a high quality diet post-hatching, and since size variation was still observed in experiments where egg size was controlled and diet was of a standard quality, it would suggest that there are likely to be multiple factors that influence growth trajectories either singly or in concert. In our experiments, we used a standard diet, and this may not have been optimal for all individuals. Diet preferences may explain the observed differences in growth rate within treatments, but do not explain the differences in the proportions of slow- and fast-growing slugs found between seasons. Shibata and Rollo’s [22] conclusion that multiple factors influence growth rates is supported by the experiments presented in this paper; egg size was not measured, but since batches were allocated to treatments at random prior to incubation, any variations in egg size would be expected to be distributed evenly amongst treatments. Whilst differences in egg size may, therefore, explain growth variation within treatments, this would not account for the significant differences observed between them.

Hatching season influenced growth at all temperatures assessed. The ambient reared slugs acted as controls and confirmed previous work that showed under field temperatures *D. reticulatum* grows faster in spring than autumn [25]. At constant temperatures of 12 and 15 °C, however, the converse was observed; growth of slugs hatching in autumn was faster than that of those hatching in spring. The 20-week monitoring period encompassed the months of May–September for spring hatching slugs and November/December–March/April for the autumn hatching slugs, depending on the hatching date. Ambient autumn temperatures were considerably lower, albeit fluctuating, than the constant treatments assessed in this study during the same monitoring period, whereas ambient spring temperatures were more similar (mean ambient temperature (± S.E.) for autumn hatching slugs = 5.6 ± 0.1 °C; spring hatching slugs = 12.7 ± 0.1 °C). It may be that the autumn hatching slugs exhibited a much greater growth response to the constant temperatures than those hatching in spring because they are more “unseasonably high” for this group. If there is something inherently different about slugs hatching from autumn laid eggs that adapts them to withstand over-wintering, then it might be expected that they would show a greater capacity to capitalise on consistently and markedly more favourable conditions than usually experienced through the winter months. In a species considered to be an r-strategist [11], the ability to respond to disturbed and changing environments is key. The finding that *D. reticulatum* is able to “step up” its growth in response to prolonged and unexpectedly mild conditions for the time of year concords with this. Therefore, whilst these results support the importance of temperature on growth, it seems that season is, independently, also influential.

Most published studies based on slugs hatching at a given point in time have shown a high positive association between growth and temperature up to an optimum, after which there is a decline in growth along with other physiological functions leading to rapid mortality [9,10,11,26,27]. Judge [12] and Clemente et al. [13], however, found the opposite; growth was greater at lower temperatures. In the experiments presented here, a positive association was confirmed for slugs hatching in autumn, but not for those hatching in spring where individuals reared at 15 °C were observed to grow significantly more slowly than those at 12 °C and ambient temperature, agreeing with Judge [12] and Clemente et al. [13]. Studies in the literature rarely state the season in which slugs hatched. It could be that those indicating a positive association between growth and temperature were based on slugs hatching in autumn, whereas those indicating a negative association used slugs hatching in spring. The studies on *D. reticulatum* by Clemente et al. [13], which showed a negative association, were carried out in the southern hemisphere in spring. The results of the current experiments, therefore, may not be contradictory, but rather help to explain this discrepancy.

Egg size may be an important determinant of whether a slug is a fast or slow grower [22], but genetic differences may also play a role. Growth rate may be controlled by a simple Mendelian trait, but it may alternatively be affected by egg fertilisation. Self-fertilisation is possible in species that normally cross-fertilise—e.g., the genus *Philomycus* [28]—although it is not the norm. Furthermore, some species lay mixed batches of eggs, fertilised by both autosperm and allosperm—e.g., *Arion* [29]. *D. reticulatum*, a normally cross-fertilising species, can lay eggs when reared in isolation, but these are said to be infertile [11,30]. In the current study, this was found not to be the case; some eggs laid by isolated slugs were fertile, and it seems, therefore, that *D. reticulatum* is also capable of both cross-fertilisation and self-fertilisation. This being the case, it may be that fast and slow growth is determined by whether the egg is fertilised by autosperm or allosperm. Preliminary studies with microsatellite markers on genetic diversity within egg batches showed that there was no evidence of multiple paternity within egg batches [31], but this is reported by other authors [32]. Further genetic studies to investigate this would be of great value.

Whilst the mortality of *D. reticulatum* under different environmental conditions has been described in the literature in terms of absolute or relative numbers, there is little work that has formally assessed survival over time and, as for growth, none that compares this between seasons. In the experiments presented here, it was found that season significantly affected the survival rate at ambient temperature and 15 °C, but not at 12 °C. Under ambient temperature, slugs survived longer in autumn than in spring, whereas at 15 °C, survival was greater in spring. The ambient results indicate that under field temperatures, *D. reticulatum* is better able to survive in cool than warm conditions. It is known that slugs possess physiological mechanisms to cope with low temperatures—for example, they can enter a state of chill coma [33]—whereby they cool to below the temperature at which freezing would normally occur (supercooling point) without becoming immobilised, and can survive freezing temperatures for longer in winter than other times of the year [34]. They are more vulnerable to warm temperatures, however, having to rely to a greater extent on behavioural adaptations to withstand extremes. Although the mean ambient temperature in spring (± S.E.), at 12.7 ± 0.1 °C, is not at the upper limit of their tolerance, this group were subject to a mean daily range in temperature that was almost double that of the autumn hatching ambient reared slugs (± 4.9 °C c.f. ± 2.8 °C). Furthermore, the maximum recorded temperature in spring was 24.1 °C compared to 14.0 °C in autumn; hence, spring hatching slugs were subjected to larger extremes of temperature, and this may also negatively impact their survival.

Hunter and Symonds [35] suggested that there are overlapping (“leapfrogging”) generations of *D. reticulatum* in temperate regions such as the United Kingdom. Under this scheme, the slug population consists of two generations separated by an interval of about nine months. In generation A, slugs hatch in autumn, over-winter and lay eggs the following spring (equivalent to the autumn hatching slugs in the current study), whereas generation B hatch in late spring and then mature and lay eggs in late autumn (equivalent to the spring hatching slugs). This hypothesis accounts for the two peaks in slug numbers in spring and autumn whilst allowing that there cannot be two complete generations in a year due to a lifespan of nine or more months from egg to adult [15]. Our results show that these two generations may not be distinct “cohorts” on the basis of age as some slugs may mature early/late and slip into the alternative cohort as suggested by South [36]. Rather, the observed autumn and spring peaks in slug numbers are possibly a response of a mixed-age population to the favourable environmental conditions at that time.

Our experiments support the hypothesis that autumn hatching slugs exhibit different growth trajectories to spring hatching slugs when reared under identical conditions suggesting that the temperature–growth relationship is more complex than previously thought. Not only does it vary with rearing temperature, but this is further modified by the hatching season. Studies of *D. reticulatum* growth, therefore, need to take into account the hatching season.

## 5. Conclusions

In conclusion we have shown that:

1. There was considerable variation in the growth of *D. reticulatum* reared under identical conditions. Slugs were either slow growers or fast growers.
2. Growth was influenced by hatching season at all rearing temperatures; at constant temperatures of 12 and 15 °C, growth was faster in autumn than spring, whereas at ambient temperature, the reverse was observed.
3. Within a season, the association between growth and temperature was low, but negative in spring; however, it was high and positive in autumn.
4. Survival was influenced by hatching season for slugs reared at ambient temperature and 15 °C and was inversely related to growth rate, but had no effect at 12 °C.
5. Within a season, the chances of survival improved at lower rearing temperatures.

## Figures and Tables

**Table 1:** Numbers of *Deroceras reticulatum* alive for the full 20-week monitoring period in each experimental treatment.

	Rearing Temperature (°C)			
Hatching Season	Ambient	12	15	Total
Spring	60	97	94	251
Autumn	94	97	96	287
**Total**	154	194	190	538

**Table 2:** Mean weight (±S.E.) (mg) at week 20 of *Deroceras reticulatum* hatching in spring and autumn, reared at ambient temperature, 12 °C or 15 °C.

	Rearing Temperature (°C)		
Hatching Season	Ambient	12	15
Spring	170.05 ± 13.62	163.04 ± 16.64	121.55 ± 12.53
Autumn	180.37 ± 7.41	671.56 ± 25.52	1002.58 ± 45.00

**Table 3:** Numbers of *Deroceras reticulatum* in the experiment up to week 20, including those that died before the numbers were reduced to 100 per treatment.

	Rearing Temperature (°C)			
Hatching Season	Ambient	12	15	Total
Spring	141	116	114	371
Autumn	108	122	134	364
**Total**	249	238	248	735

**Table 4:** Mean survival times (±S.E.) (weeks) for *Deroceras reticulatum* hatching in spring and autumn and reared at ambient temperature, 12 °C or 15 °C (weeks 0–20).

	Rearing Temperature (°C)		
Hatching Season	Ambient	12	15
Spring	12.65 ± 0.68	17.26 ± 0.60	17.12 ± 0.60
Autumn	17.81 ± 0.55	16.51 ± 0.66	15.12 ± 0.70
